# Utilizing Social Media Data for Psychoanalysis to Study Human Personality

**DOI:** 10.3389/fpsyg.2019.02596

**Published:** 2019-11-15

**Authors:** Frank Emmert-Streib, Olli Yli-Harja, Matthias Dehmer

**Affiliations:** ^1^Predictive Society and Data Analytics Lab, Faculty of Information Technology and Communication Sciences, Tampere University, Tampere, Finland; ^2^Faculty of Medicine and Health Technology, Institute of Biosciences and Medical Technology, Tampere University, Tampere, Finland; ^3^Faculty for Management, Institute for Intelligent Production, University of Applied Sciences Upper Austria, Steyr, Austria; ^4^Department of Mechatronics and Biomedical Computer Science, University for Health Sciences, Medical Informatics and Technology (UMIT), Hall in Tirol, Austria; ^5^College of Artificial Intelligence, Nankai University, Nankai, China

**Keywords:** psychoanalysis, computational social science, data science, privacy, psychology

## Abstract

Social media data, for instance from Twitter or Facebook, provide a new type of data that consist of a mixture of text, image and video information. From a scientific point of view, the capabilities of this type of data from such microblogs are not well explored and to date it is largely unknown what principal knowledge can be extracted thereof. In this paper, we present a discussion of the capabilities of data from microblogs for performing a psychoanalysis. This could allow an analysis of the human personality of individual users. Such prospects raises serious concerns regarding the privacy of users of social media platforms.

## 1. Introduction

In recent years, social media generated widespread interest in almost all fields of science including psychology (Correa et al., 2010; Bolton et al., 2013; Kalampokis et al., 2013; Schoen et al., 2013). Of particular interest is, how to utilize such data for making predictions (Emmert-Streib et al., 2018). For instance, data from Twitter or Facebook have been used for predicting consumer behavior (Ringelhan et al., 2015), opinion flow in communities (Wu et al., 2014), account classification (Chu et al., 2010, 2012; Dickerson et al., 2014), conflicts among friends (Liu and Weber, 2014), demographics of users (Culotta et al., 2015), mental health of users (Guntuku et al., 2017), tourism (information search and decision-making behaviors) (Zeng and Gerritsen, 2014), stock market behavior (Bollen et al., 2011; Siganos et al., 2014), national and international election results (Tumasjan et al., 2011; Alonso and Vilares, 2016), word-of-mouth (WOM) or consumer reviews (Zhang et al., 2012) and box-office revenue of movies (Asur and Huberman, 2010). Furthermore, also in psychology related problems have been studied, e.g., prediction models investigating the emotional constitution of people (Fernandez et al., 2012; Kross et al., 2013; Ortigosa et al., 2014), mental health (Guntuku et al., 2017) or the personal traits and characters of individuals (Kosinski et al., 2013).

Interestingly, the principle theoretical capabilities of data provided by Twitter or Facebook, in general called microblogs (Zhao and Rosson, 2009), are largely unexplored. Instead, the above mentioned applications focus on heuristic methods and practical problems without a deeper justification for the applicability of the used approaches demonstrating the usefulness of such data in particular application domains for individual case studies. In contrast, in this paper, we present a generic perspective on the theoretical capabilities of microblogs of social media, e.g., provided by Twitter or Facebook, for psychology. Specifically, we present a discussion of the principle capabilities that the information provided by such microblogs offer and argue that it is suitable for performing a psychoanalysis (Erdelyi, 1985) for studying the human personality of individual users. Such prospects could have far reaching consequences for the privacy rights of users of microblogging platforms because they imply a public transparency with respect to the most intimate information of individuals, namely their personality.

This paper is organized as follows. In the next section, we present background information from psychoanalysis as far as it is needed for our analysis. Then we present numerical results and discuss implications for the privacy rights of users. This paper finishes with concluding remarks.

## 2. Basics From Psychology

We start our analysis by providing a brief review of psychoanalysis and related methods as far as needed for our analysis.

The term *psychoanalysis*, sometimes also called psychodynamics, was coined by Sigmund Freud (Freud, 1916, 2014), who conceived, developed, and applied this method. The purpose of psychoanalysis is threefold:

- First, it is a method for investigating and understanding the functioning of the mind.
- Second, it is a form of treatment for mental illness.
- Third, it is a branch of psychological or behavioral science.

A key element of this method is the importance in mental life of unconscious mental phenomena, that is, of mental processes of which the subject himself has no conscious knowledge or awareness. This may include an individual's vulnerabilities, motives, tensions, impulses, guilt, fantasies, or urges. One of the goals of psychoanalysis is to help the patient to develop insight into his/her unconscious processes.

An early method practiced by Freud was called *Free Association* (Aron, 1996; Kris, 2013). This method replaced hypnosis in Freud's therapy. It consists of gathering the free association provided by the patient during the therapy sessions. These associations point to the inner conflicts and repressed drives (Triebe) of the patient that made the symptoms. Later modifications of psychoanalysis have been developed, e.g., psychoanalytic psychotherapy that nowadays is much more widely practiced. However, all such developments are based on free association forming the golden standard of the psychoanalytic therapy. We would like to add that *transference* (Klein, 2017), i.e., the unconscious projection of the feelings of patients about their parents, is an important part of such a therapy.

At present, psychodynamics is an evolving multi-disciplinary field which analyzes and studies human thought processes, response patterns, and their influences. The purpose of this research is to provide insights into the following:

- Understanding and anticipating the range of specific conscious and unconscious responses to specific perceptions (sensory input in the form of images, colors, textures, sounds, etc.).
- Utilizing the communicative nature of movement and primal physiological gestures to affect and study specific mind-body states.
- Examining the general capacity for the mind and senses to directly affect physiological response and biological change.

Interestingly, it has been realized by Freud that the verbal information provided by the patient to the therapist during the therapy sessions can be transcribed into text, which then contains an equal amount of information. The reason for this is that a key element of a therapy sessions is that the therapist does not *actively guide* the patient in a certain direction, but the patient talks freely about any topic only internally guided by her/his own unconscious mind. Hence, this corresponds more to a monolog rather than a dialog between two people. A consequence of this is that psychoanalysis has been applied to literature which is called *psychoanalytic literary criticism* (Booker, 1996; Holland, 2000; Ellmann, 2014). The purpose of this application is to conduct a psychoanalysis about an author of a book or even a fictional character ocurring within a book. Overall, this provides us with a practical connection between psychology and the data provided by microblogs.

## 3. Quantifying Tweets: Converting Tweets Into Book Pages

Data from microblogging platforms like Twitter or Facebook are very heterogeneous with respect to individual users. The reason for this is that the usage profiles of the users varies widely. For instance, there are users who registered at these services but never participate at all in any form. On the other hand, there are users who are very active posting thousands of messages. From this it is clear that the analysis capabilities of such microblogging platforms are not generic but highly user-specific.

In the following, we will formulate a hypothesis that is limited to the active users only. Specifically, we hypothesize that if the information provided by literature is adequate in order to perform a psychoanalysis of an author, or a fictional character of a book, then the information provided by microblogs allows a similar analysis.

Certainly, for our hypothesis, similar restrictions apply as for psychoanalytic literary criticism meaning that not every piece of literature provides adequate information. For instance, very short books or certain literature genres may not be suitable. Similarly, Twitter or Facebook users posting only very few tweets or posts are not suitable for such an analysis. However, for users writing many messages such an analysis can be feasible. In order to quantify this argument we provide numerical results for Twitter.

**Figure 1:**
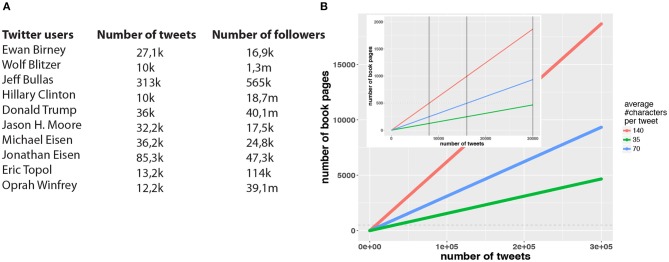
**(A)** An overview of active Twitter users. Shown is information about the number of tweets and the number of followers. **(B)** The number of tweets is converted into the number of equivalent book pages. The three curves correspond to different tweet lengths, as expressed by the average number of characters (140 characters red line, 70 characters blue line and 35 characters green line) these tweets contain. The inlay shows a magnification of the results and the three vertical lines correspond to the intersections between the three curves and 500 book pages, serving as a reference value (horizontal dashed line).

In Figure 1A an overview of active Twitter users is shown providing their number of tweets and the number of their followers. In Figure 1B we show a conversion of the number of tweets into the number of equivalent book pages. For this conversion we are assuming that the average English word consists of 4.5 characters and an average book page contains 500 words. The three curves we are showing in Figure 1B correspond to three different models, whereas each model assumes a different average tweet length, as expressed by the average number of characters (140 characters: red model, 70 characters: blue model and 35 characters: green model) which these tweets contain. The inlay shows a magnification of the results and the three vertical lines correspond to the intersections between the three curves and 500 book pages, serving as a reference value (horizontal dashed line). The intersection points are occuring at 8,000, 16,000, and 30,000 tweets. Comparing these values with the number of tweets shown in Figure 1A one can see that no matter what average number of characters per tweet is assumed, all Twitter users tweeted about 500 book pages or more (for instance Jeff Bullas tweeted an equivalent of 4,666, 9,333, 18,666 book pages; corresponding the three different models).

In addition to this textual information, it is known that about 40% of all tweets include an image. That means there is an additional modality of information available corresponding, e.g., to 3,200, 6,400, and 12,000 images for the three intersection points discussed above. For ordinary literature, this type of information is usually absent. Furthermore, Twitter allows users to link videos to tweets. This gives indeed a third information modality that enriches the textual information provided by the tweets.

As we have already mentioned, Freud claimed that a patient should talk freely about any topic internally guided by her/his unconscious mind, we omit semantic aspects of the tweets within our analysis. However, in principle, each sentence a patient says can be semantically quantified (Rieger, 1989; Tuldava, 1995) and a semantic function could be defined by composing the elementary semantic expressions, see, e.g., Manes and Arbib (1986).

Furthermore, we would like to note that Twitter (but also Facebook) offers the possibility to reply to tweets. This could be interpreted as a dialog between the owner of a tweet and a reader in analogy to the interaction of a patient with her/his therapist. Due to the fact that there is no obligation of the tweet owner to engage in such a dialog this is similar to the non-guiding comments/questions of a therapist in a therapy session. Hence, the free flow of thought processes of a Twitter user is not suppressed nor perturbed in a profound way different from a therapy session.

Taken together, this discussion shows there exist very active Twitter users that produce information which is comparable to the information used for a psychoanalytic literature criticism. Hence, for such users a similar psychoanalysis should be feasible.

## 4. Beyond Sentiment Analysis

It is clear that a psychoanalysis is profoundly more difficult than a sentiment analysis, which merely tries to determine emotional or opinion categories (Pang and Lee, 2008; Agarwal et al., 2011). However, a sentiment analysis can be part of a psychoanalysis by contributing toward such an assessment.

**Figure 2:**
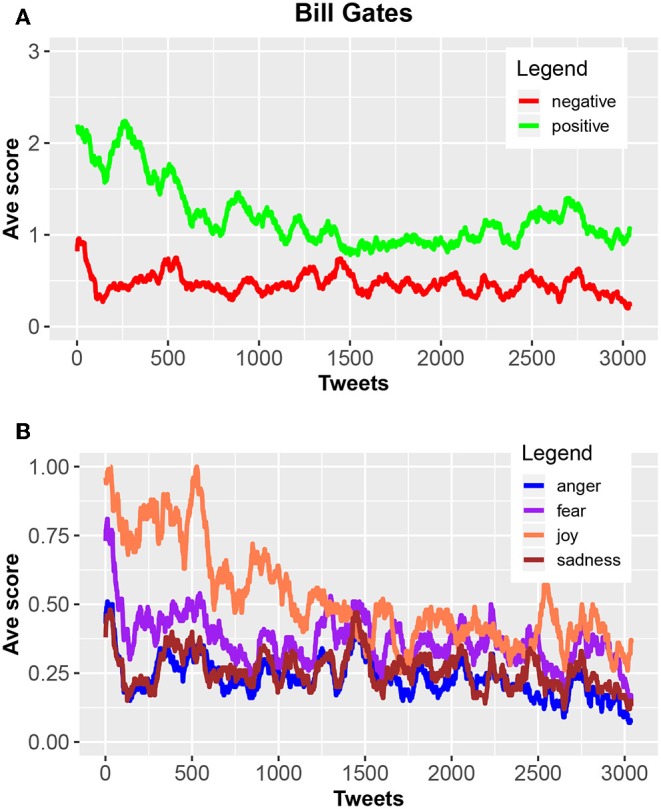
Sentiment analysis of tweets from Bill Gates. **(A)** A categorization in positive (green) and negative (red) tweets. **(B)** Four subcategories of the tweets. All results have been averaged over a sliding window of 100 tweets.

An example of a sentiment analysis for the tweets from Bill Gates is shown in Figure 2. For this analysis, we used the NRC sentiment dictionary (Mohammad and Turney, 2010) to identify different emotional categories. The shown results have been averaged over a sliding window of 100 tweets to obtain smoother curves. Overall, since his registration in June 2009 he contributed over 3,000 tweets.

The most interesting result for informing a psychoanalysis could be the change in the temporal evolution. Specifically, the distance between the number of negative and positive tweets but also between anger, fear, joy and sadness, decreases over time. From Figures 2A,B one can see, that this change happened after about 1,500 tweets. Hence, from such an analysis one could identify different temporal regimes to be used for a psychoanalysis because it is plausible that human personality is not constant over time but changes. In the case of Bill Gates, one could argue that one finds two such regimes.

## 5. An Example: Psychological Targeting

In a study by Matz et al. (2017), the effect of *psychological targeting* on advertising has been studied. The authors demonstrated that by first predicting the personality of social media users and then using this for psychologically tailored advertising on Facebook—called psychological targeting—has more persuading appeal. Specifically, differences in the consumer buying behavior have been recorded assessing the decision making of the individuals. Their field experiment involved over 3.5 million individuals and hence provides an impressive hint on the power of knowledge about human personality and how it could be exploited by third parties.

The success of this study depends to a considerable extend on the quality of the predicted personality of users. Previous studies have address this problem based on data from personal websites, blogs, Twitter and Facebook (Marcus et al., 2006; Golbeck et al., 2011; Park et al., 2015). The quality of such studies is often difficult to assess especially in isolation. However, given the decision making component of the field experiment by Matz et al. (2017) allows an indirect assessment via the observed differences in user behavior.

We would like to emphasize that the study by Matz et al. (2017) did not claim that every individual can be targeted successfully nor did this study claim to be able to infer all information about human personality (focus was placed on extraversion and openness to experience). Instead, they showed *in average* individuals can be targeted based on information available. Hence, even incomplete information is sufficient to improve upon traditional mass advertising. From a data science perspective this is plausible because a similar approach is currently used in precision medicine to improve upon traditional medicine (Chen and Snyder, 2013; Emmert-Streib and Dehmer, 2018).

## 6. Discussion

The results from our discussion is alarming regarding the privacy rights of individuals. The reason for this may be clarified by an example. Suppose a patient is in a psychiatric treatment. Then information about this treatment is strictly confidential and not accessible to a third party, e.g., the public, without the consent of the patient. On the other hand, our discussion shows that, certain users of microblogging platforms, are in risk of providing indirectly access to such information in the form of their blogging contents. In case they don't mind for such an information to be public, there is no problem, however, we doubt that most users are even aware of such a threat and would not consent to the public distribution of this intimate information.

We think that information about the human personality of individuals is among the most sensitive information one can obtain about someone. For this reason, our results suggest that users of microblogging platforms should be made aware of such threats so they can decide if they are willing to provide such information to the public. As examples, where such information could be exploited we just mention future employers or insurance companies.

Finally, we would like to remark that our arguments above are meant in a probabilistic manner. That means, instead of absolute statements like “always” our arguments hold with a certain probability for specific individuals. This refers also to entities as “human personality” which may not be identifiable to 100% but certain aspects of it. However, as the example of psychological targeting discussed above has shown, these aspects may be sufficient for an exploitation by third parties.

## 7. Conclusion

Overall, the above numerical analysis showed that there are active Twitter users that generate textual information corresponding to an equivalent of 500 or more book pages and extreme examples, as Jeff Bullas, generate even thousands or ten thousands of such pages. In addition, this textual information is complemented by thousands of images and videos providing potentially rich information about the users. Hence, the output of such Twitter users could be analyzed by the psychoanalytic literary criticism (Booker, 1996; Holland, 2000; Ellmann, 2014) enabling a psychoanalysis of the Twitter users.

Of course, quantity does not imply quality but the more information an individual provides, e.g., in the form of tweets, the more likely it is that this contains quality information that allows a prediction of the users's personality. Even if this does only allow to obtain partially information of human personality this may be enough for certain practical applications, as psychological targeting, that are not necessarily in the individual's interest.

In summary, the above arguments support our hypothesis by demonstrating that quantitatively as well as qualitatively the information provided by Twitter, for exclusive users, is at least equivalent to information provided by adequate literature enabling a psychoanalytic literary criticism. This enables a computational psychology approach to study human personality *in average* using data from social media.

## Author Contributions

FE-S conceived the study. All authors contributed to the writing of the manuscript and approved the final version.

### Conflict of Interest

The authors declare that the research was conducted in the absence of any commercial or financial relationships that could be construed as a potential conflict of interest.
